# Supplementary material for: Twenty years of tuberculosis-driven selection shaped the evolution of the meerkat major histocompatibility complex
Source: Nat Ecol Evol. 2025 Aug 25;9(11):2161–72. doi: 10.1038/s41559-025-02837-x (PMC12592209; doi:10.1038/s41559-025-02837-x)
Supplement: Supplementary file 1 — Supplementary Material and Methods, Results, References, Figs. 1–5 and Tables 1–3 and 5–9. [file 41559_2025_2837_MOESM1_ESM.pdf]

nature ecology & evolution

Supplementary information https://doi.org/10.1038/s41559-025-02837-x

Twenty years of tuberculosis-driven selection shaped the evolution of the meerkat major histocompatibility complex

In the format provided by the authors and unedited

Table of contents

Supplementary Material and Methods
- *Library Preparation*
- *Quality filtering of paired-end reads and MHC allele calling*
- *Manual MHC class II gene annotation*
  - *Transcriptome assembly*
  - *Manual MHC class II annotation and allele mapping*

Supplementary Results
- *MHC class II gene annotation*
- *Biological, socio-ecological and environmental factors impact TB susceptibility, progression, resilience, lifetime reproductive success and survival*

Supplementary References

Supplementary Figures

Supplementary Tables

Supplementary Data files

Supplementary Material and Methods

Library Preparation

Tissue samples were collected following a standardized Kalahari Meerkat Project (KMP) protocol[1,2]. DNA was extracted at the Institute of Evolutionary Biology, School of Biological Sciences (University of Edinburgh, UK) and the Department of Evolutionary Biology and Environmental Studies (University of Zurich, Switzerland), and aliquots were transported on ice to the Institute of Evolutionary Ecology and Conservation Genomics (Ulm University, Germany), and stored at -20°C.

Following MHC primer validation, we followed standard in-house procedures for high-throughput sequencing of exon 2 of the MHC class II DRB gene(e.g.,[3]). Briefly, MHC-DRB exon II was amplified via polymerase chain reaction (PCR) in 10 µl reactions, containing 2.4 µl purified water, 5.0 µl AmpliTaq Gold™ 360 Master Mix (Applied Biosystems, Darmstadt, Germany), 1.0 µl GC-rich buffer and, 300 nM of both forward (CS1_Crcr_MHC2F CCTGTSYCCACAGCACATTTCYT) and reverse primer (CS2_Crcr_MHC2R GCTCAMCTCGCCGSTGCAC) and 1 µl of DNA template. Amplification protocols were denaturation at 95°C for 10 min, followed by 33 cycles of denaturation at 95°C for 30 s, annealing at 56°C for 30 s, and elongation at 72°C for 60 s, with a final elongation step at 72°C for 60 s. PCR success was confirmed by agarose gel electrophoresis. Some samples collected early in the study period (N= 133) repeatedly failed in first PCRs, but could be PCR amplified after purification with the ZR-96 DNA Clean & Concentrator Kit following manufacturer's instructions (Zymo Research, Freiburg, Germany).

Barcoding PCRs were performed using 20 µl preparations containing 1.5 µl purified water, 10 µl AmpliTaq Gold™ 360 Master Mix, 2.0 µl GC-rich buffer, 2.5 µl PCR product and 4.0 µl Illumina adapter with individual barcodes (Access Array™ System for Illumina Sequencing Systems, Standard BioTools, South San Francisco, USA), and amplified by 10 min denaturation at 95°C, followed by 10 cycles of denaturation at 95°C for 30 s, annealing at 60°C for 30 s, elongation at 72°C for 60 s, and final elongation at 72°C for 10 min. To remove artifacts and excess chemicals, samples were purified using a NucleoMag® NGS Clean-up and Size Select Kit (Macherey-Nagel, Düren, Germany) on a GeneTheatre™ pipetting robot (Analytik Jena, Jena, Germany) according to the manufacturer's guidelines, followed by quality assessment via capillary electrophoresis on a QIAxcel® Advanced System (QIAGEN, Hilden, Germany). For normalization, we measured DNA concentration in the samples using the QuantiFluor® dsDNA System (Promega, Madison, USA) on a TECAN Infinite F200 PRO® plate reader (Tecan, Männedorf, Switzerland) and normalized samples to include 60 ng of indexed amplicon using the Illumina MiSeq Denature and Dilute Libraries Guide (version 15039740-10). The library was loaded at 8 pM on a MiSeq flow cell (Illumina MiSeq Reagent Kit V2) with 5 % PhiX sequencing control V3 spiked in and paired-end sequencing was performed over 2 x 250 cycles on the Illumina MiSeq.

High throughput sequencing of meerkat Susu-MHC-DRB exon 2 was performed for 1567 individuals on an Illumina MiSeq platform, across six Illumina runs, with 360 technical replicates (up to four technical replicates per individual) and 23 negative controls. This generated 80,102,878 raw reads (42,383 ± 18,656 raw reads per sample), with 56,238,309 reads (29,756 ± 13,288 per sample) retained after quality filtering. All negative samples had less than 12,000 raw reads, out of which less than 30 reads had blast hits. There was no indication for a link between the number of retained reads and number of called MHC alleles.

Quality filtering of paired-end reads and MHC allele calling

Quality assessment of the paired-end reads generated by Illumina and subsequent MHC allele calling was performed using the ACACIA (Allele Calling Procedure for Illumina Amplicon Sequencing data) bioinformatic pipeline[4] under default settings, excluding sequences represented by less than 1 % of the reads of the sample and 100 blasted reads. For the internal Blast reference, sequences of Mammalian-DRB exon II were extracted from NCBI on the 31st of October 2019 (Search terms: "Mammalia"[Organism]) AND MHC-DRB [All Fields]) AND alive[prop]), generating a FASTA file including 219 sequences. For final MHC genotype assignment following the bioinformatic pipeline, we manually excluded samples with less than 10.000 reads, MHC alleles with a mean proportion of reads of less than 5 % [5], MHC alleles occurring in less than five individuals, and MHC alleles with more than 80 % of occurrences in only one Illumina run. Assessing 360 technical replicates of 170 individuals, we found repeatability at individual level (complete match) at 79.4% and repeatability at MHC allele level at 96.1%. For individuals with technical replicates, we retained the sample with the highest read count, including all alleles replicated at least twice. Following manual curation of the quality checked Illumina-output yielded a final dataset of 50,552,430 reads (32,220 ± 12,254 reads per sample) for samples of 1,567 individuals.

We detected 43 distinct meerkat MHC-DRB alleles of 247 bp in length. 37 sequences had a continuous open reading frame starting at position 2, whereas six (DRB*02, *04, *05, *19, *23 and *28) showed a premature stop codon at amino-acid position 82 and were thus presumed non-functional and excluded from subsequent analyses. The functional MHC alleles DRB*01, DRB*41 and DRB*56 as well as the presumed non-functional alleles contained a 9 bps long insertion at position 231, resulting in translations into sequences of 82 and 85 amino acids, respectively. Only DRB*41 and DRB*56 translated into the same amino-acid sequences. The 37 alleles with open reading frame clustered into 12 functionally distinct supertypes (Supplementary Table 7) and could be assigned to 16 putatively functional haplotypes (Supplementary Table 8). Notably, non-functional MHC alleles were included for haplotyping as they were being passed on to offspring, but were not included in subsequent analyses. Analyses were only carried out for functional alleles and haplotypes.

Manual MHC class II gene annotation

Transcriptome assembly

We undertook reference guided transcriptome assembly of 14 publicly available meerkat tissues from two individuals (Supplementary Table 3). Raw fastq files were quality checked with fastqc v0.11.8[6]and summarised with multiqc v1.15.dev0[7]. Raw fastq files were then trimmed using trimmomatic v0.39[8] with the following parameters; TruSeq3-PE.fa:2:30:10 SLIDINGWINDOW:4:5 LEADING:5 TRAILING:5 MINLEN:25. Paired trimmed files were then aligned to the meerkat reference genome (GCF_006229205)[9,10] using hisat2 v2.1.0[11] with the downstream-transcriptome-assembly flag, resulting sam files were sorted and converted to bam using samtools v1.17[12]. Output bam files were converted to gtf using stringtie v2.1.6[13], with 14 tissue specific transcriptomes merged with stringtie merge.

Manual MHC class II annotation and allele mapping

We undertook manual MHC class II annotation by performing blastn[14] searches against the meerkat reference genome with default parameters and human query sequences downloaded from The IPD-IMGT/HLA Database[15,16]. Blast hits for each exon were manually inspected in IGV v2.17.2[17] along with aligned transcripts to determine correct splice sites and open reading frame. All allele sequences identified were later used as query sequences for blastn against the meerkat reference genome

(GCF_006229205) with default parameters. The best blast hit for each allele sequence was retained and compared to manually annotated class II genes to identify which locus the allele occurred in.

Supplementary Results

MHC class II gene annotation

Manual annotation of class II MHC genes identified a full-length single copy of DRA, DQB, DOA, DOB, DMA, DMB, DPA and DPB on scaffold NC_043706.1 (Supplementary Table 4). We were only able to identify fragments of each DQA exon, with exon one present in the main MHC class II region on scaffold NC_043706 and other fragments present across NW_021870370.1, NW_021877437.1, NW_021891667.1 and NW_021891698.1, in total we located two copies of exon 2, 3 and 4 and a single copy of exon 1 indicating DQA present in either one or two copies in meerkats. However, it is noteworthy that DQA is fragmented in the current reference genome, which is not surprising given the technologies used to generate the assembly[18] We identified a single full length and a single pseudogene of DRB on NC_043706.1 and also on NW_021859663.1, we also identified additional exon two sequences on NW_021880693.1, NW_021885817.1. The two fragments of exon 2 each contained a stop codon, indicating there may be a total of two full length DRB genes (Supplementary Table 5). Our difficulty in annotating MHC genes in the meerkat is not surprising given the method of assembly, short read sequencing and scaffolding using HiC[18]. All full-length class II genes on NC_043706 and the DRB gene on NW_021859663.1 showed evidence of expression across multiple tissue types, giving evidence that these are all functional MHC genes (Supplementary Table 5). Additionally, both DRB pseudogenes were not expressed providing evidence for their function as pseudogenes (Supplementary Table 4 + 5). The fragments of DQA and DRB exon 2 also showed evidence of expression across multiple tissue types.

All 43 DRB exon 2 alleles had blast hits to either exon 2 of DRB1, DRB2 or the exon 2 fragments on NW_021880693.1, NW_021885817.1. The six alleles that contained stop codons (DRB*02, *04, *05, *19, *23 and *28) hit best to DRB exon 2 on either NW_021880693.1 or NW_021885817.1. Interestingly, there are other alleles (DRB*01, *13, *41 and *56) which also have the best hits to NW_021880693.1 and NW_021885817.1 but do not contain stop codons, indicating the potential that these do encode for functional genes. Nevertheless, a mutation that results in a premature stop codon is evident in meerkats. All other alleles mapped to exon 2 of either DRB1 or DRB2, with no alleles hitting to either pseudogene or other MHC class II genes, highlighting these are all functional.

Biological, socio-ecological and environmental factors impact TB susceptibility, progression, resilience, lifetime reproductive success and survival

Throughout our analyses, we found effects of biological, socio-ecologic and environmental factors on meerkat-TB dynamics. Here, we report the results for the baseline models, including number of DRB-alleles or haplotype heterozygosity and biological, socio-ecological and environmental factors, for all variables that were consistently retained in the best models during model selection and significant throughout all models run with the respective dataset. Detailed results for the models including further MHC measures are reported in Supplementary Tables 9-14. All p-values are fdr-adjusted.

We found a significant positive impact of year on TB susceptibility in both the full (Estimate: 1.02, CI: 1.30-0.75, p-value < 0.001; Supplementary Table 9a) and haplotyped dataset (Estimate: 1.30, CI: 1.74-

0.86, p-value < 0.001, Supplementary Table 9c), suggesting the chance of developing clinical signs of TB increases with time. Subordinate individuals were consistently less likely to progress to clinical signs than dominants (e.g., Estimate: -0.96 CI: -1.28- -0.65, p-value < 0.001), potentially due to their markedly shorter average life expectancy compared to dominant individuals, increasing the risk of infected subordinates dying before the onset of clinical TB.

Subordinate individuals that did develop clinical signs had a lower TB resilience, i.e., they died of TB earlier than their dominant conspecifics (e.g., Estimate: -0.22, CI: -0.30- -0.14, p-value < 0.001, Supplementary Table 11a,). Males were marginally less resilient than females (e.g., Estimate: -0.09, CI: -0.16- -0.01, p-value < 0.05). Individuals were more resilient over time, indicated by a significant positive effect of year (e.g., Estimate: 0.18 CI: 0.07-0.28, p-value < 0.01). But given the negative effect of the quadratic term of year (e.g., Estimate: -0.25 CI: -0.43- -0.07, p-value< 0.05) resilience actually decreased towards the final years of the study. These effects were not recovered reduced dataset (Supplementary Table 11c).

Lifetime reproductive success was primarily predicted by age (e.g., Estimate: 5.03, CI: 4.30- 5.76, p-value <0.001; Supplementary Table 13a), which means that individuals that live longer lives reproduced more. As expected, subordinates reproduced less (e.g., Estimate: -0.95, CI: -1.45- -0.46, p-value <0.001). LRS also decreased with time (e.g., Estimate: -0.73, CI: -1,02- -0.44, p-value <0.001), in line with previous findings of reduced fecundity over time[19]. In the reduced dataset, a marginally positive effect of individual H_{exp} was found (e.g., Estimate: 1.19, CI:0.04- 2.34, p-value<0.05; Supplementary Table 13)

TB progression increased and later decreased over the course of the study, indicated by a significant negative effect of the quadratic term of year in the full dataset (e.g., Estimate: -1.49, CI: -2.14- -0.85; p-value < 0.001; Supplementary Table 10a). Additionally, mean maximum temperature was significantly positively linked to TB progression (e.g., Estimate: 0.74, CI: 0.37- 1.11, p-value < 0.001), mirroring findings that TB and climate change exacerbating each other[20]. We also found a small, but consistent effect of social group size, with individuals inhabiting larger groups having a marginally lower risk of progressing to clinical TB (e.g., Estimate: -0.07, CI: -0.09- -0.06, p-value < 0.001).

Finally, survival likelihood decreased with age, with older individuals having an almost 10fold higher mortality risk (e.g., Estimate: 2.49, CI: 2.24-2.73, p-value < 0.001; Supplementary Table 12a). The effect of social status was slightly smaller, with higher mortality risk for subordinates (e.g., Estimate: 1.963, CI: 1.78- 3.25, p-value < 0.001,), mirroring previous results[21]. Clinical signs of TB strongly predicted survival (e.g., Estimate: 1.68, CI: 1.11-2.25, p-value < 0.001), with increased mortality risk of TB-infected individuals as the study progressed, indicated by a interaction between TB and the quadratic term of year (e.g., Estimate: 1.39, CI: 0.33-2.45 p-value < 0.05). This aligns with the reduced resilience in later study years. Meerkats were likely to survive years with less rainfall (Estimate: -0.68, CI: -0.81- -0.56, p-value< 0.001), and were most likely to survive in the wet-cool seasons (April to June) than in any other season (Supplementary Table 12). Lastly, individuals inhabiting larger groups benefiting from a marginally lower mortality (e.g., Estimate: -0.04, CI: -0.05- -0.03, p-value < 0.001).

Supplementary References

1. Nielsen, J. F. *et al.* Inbreeding and inbreeding depression of early life traits in a cooperative mammal. *Mol Ecol* **21**, 2788–2804 (2012).
2. Spong, G. F., Hodge, S. J., Young, A. J. & Clutton-Brock, T. H. Factors affecting the reproductive success of dominant male meerkats. *Mol Ecol* **17**, 2287–2299 (2008).
3. Schmid, D. W. *et al.* MHC class II genes mediate susceptibility and resistance to coronavirus infections in bats. *Mol Ecol* **32**, 3989–4002 (2023).
4. Gillingham, M. A. *et al.* A novel workflow to improve genotyping of multigene families in wildlife species: An experimental set-up with a known model system. *Mol Ecol Resour* **21**, 982–998 (2021).
5. Kaesler, E. *et al.* Shared evolutionary origin of major histocompatibility complex polymorphism in sympatric lemurs. *Mol Ecol* **26**, 5629–5645 (2017).
6. Andrews, S. FastQC. A quality control analysis tool for high throughput sequencing data. (2010).
7. Ewels, P., Magnusson, M., Lundin, S. & Käller, M. MultiQC: summarize analysis results for multiple tools and samples in a single report. *Bioinformatics* **32**, 3047–3048 (2016).
8. Bolger, A. M., Lohse, M. & Usadel, B. Trimmomatic: a flexible trimmer for Illumina sequence data. *Bioinformatics* **30**, 2114–2120 (2014).
9. Dudchenko, O. *et al.* De novo assembly of the Aedes aegypti genome using Hi-C yields chromosome-length scaffolds. *Science* **356**, 92–95 (2017).
10. Dudchenko, O. *et al.* The Juicebox Assembly Tools module facilitates de novo assembly of mammalian genomes with chromosome-length scaffolds for under $1000. 254797 Preprint at https://doi.org/10.1101/254797 (2018).
11. Kim, D., Paggi, J. M., Park, C., Bennett, C. & Salzberg, S. L. Graph-based genome alignment and genotyping with HISAT2 and HISAT-genotype. *Nat Biotechnol* **37**, 907–915 (2019).
12. Danecek, P. *et al.* Twelve years of SAMtools and BCFtools. *Gigascience* **10**, giab008 (2021).

13. Pertea, M. *et al.* StringTie enables improved reconstruction of a transcriptome from RNA-seq reads. *Nat Biotechnol* **33**, 290–295 (2015).

14. Altschul, S. F., Gish, W., Miller, W., Myers, E. W. & Lipman, D. J. Basic local alignment search tool. *Journal of Molecular Biology* **215**, 403–410 (1990).

15. Barker, D. J. *et al.* The IPD-IMGT/HLA Database. *Nucleic Acids Res* **51**, D1053–D1060 (2023).

16. Robinson, J., Barker, D. J. & Marsh, S. G. E. 25 years of the IPD-IMGT/HLA Database. *HLA* **103**, e15549 (2024).

17. Robinson, J. T. *et al.* Integrative genomics viewer. *Nat Biotechnol* **29**, 24–26 (2011).

18. Peel, E. *et al.* Best genome sequencing strategies for annotation of complex immune gene families in wildlife. *GigaScience* **11**, giac100 (2022).

19. Paniw, M., Maag, N., Cozzi, G., Clutton-Brock, T. & Ozgul, A. Life history responses of meerkats to seasonal changes in extreme environments. *Science* **363**, 631–635 (2019).

20. Paniw, M. *et al.* Higher temperature extremes exacerbate negative disease effects in a social mammal. *Nat. Clim. Chang.* **12**, 284–290 (2022).

21. Cram, D. L. *et al.* Rank-related contrasts in longevity arise from extra-group excursions not delayed senescence in a cooperative mammal. *Current Biology* **28**, 2934-2939.e4 (2018).

Supplementary Figures

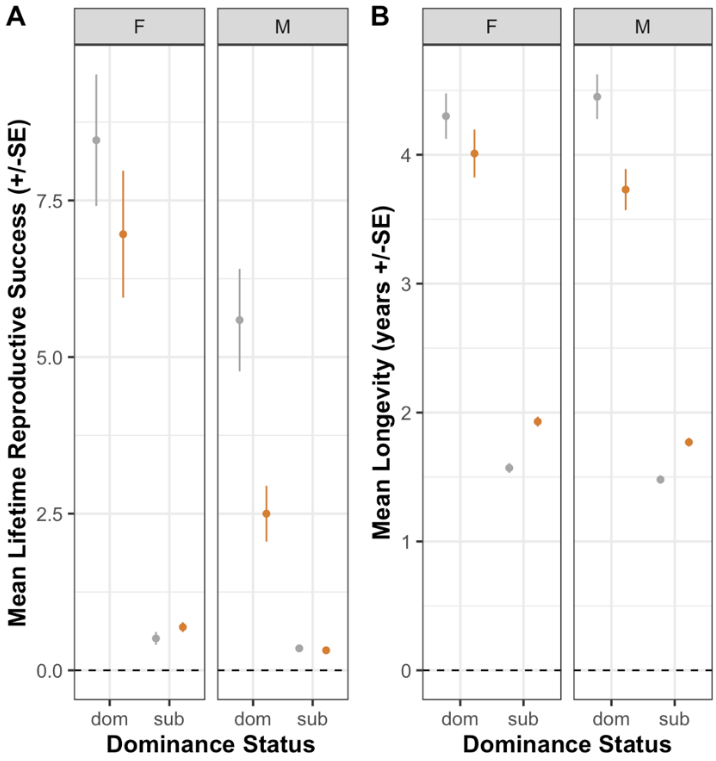

Supplementary Figure 1. Comparison of (A) mean lifetime reproductive success (± standard error) and (B) mean longevity (± standard error) recorded for the different sexes (F/M), social hierarchy (dominant/subordinate) and whether meerkats were infected with TB (orange) or uninfected (grey).

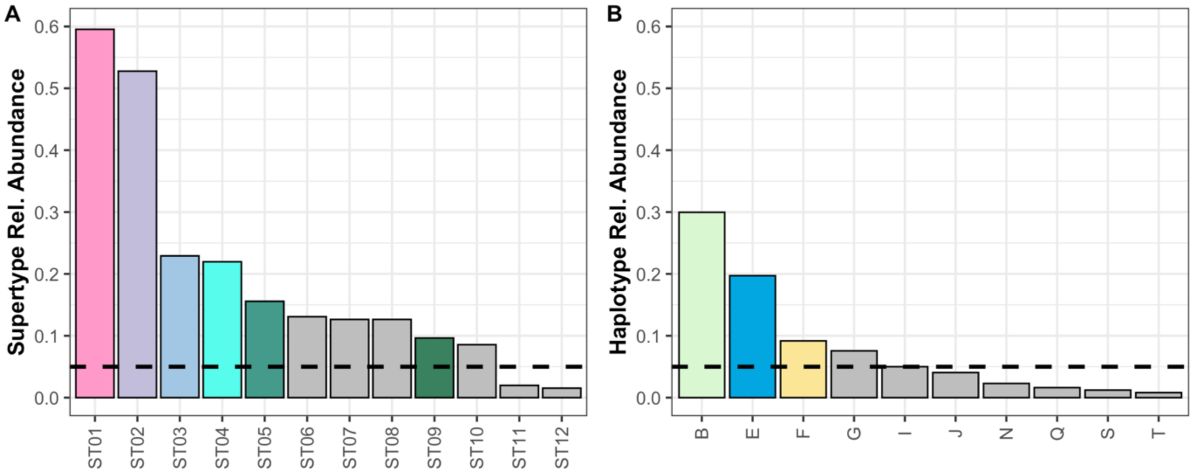

Supplementary Figure 2. (A) Relative abundance of MHC DRB supertypes and (B) haplotypes. The dashed line indicates the 5% threshold. Colourful supertypes were also found in at least ten and haplotypes in at least five individuals.

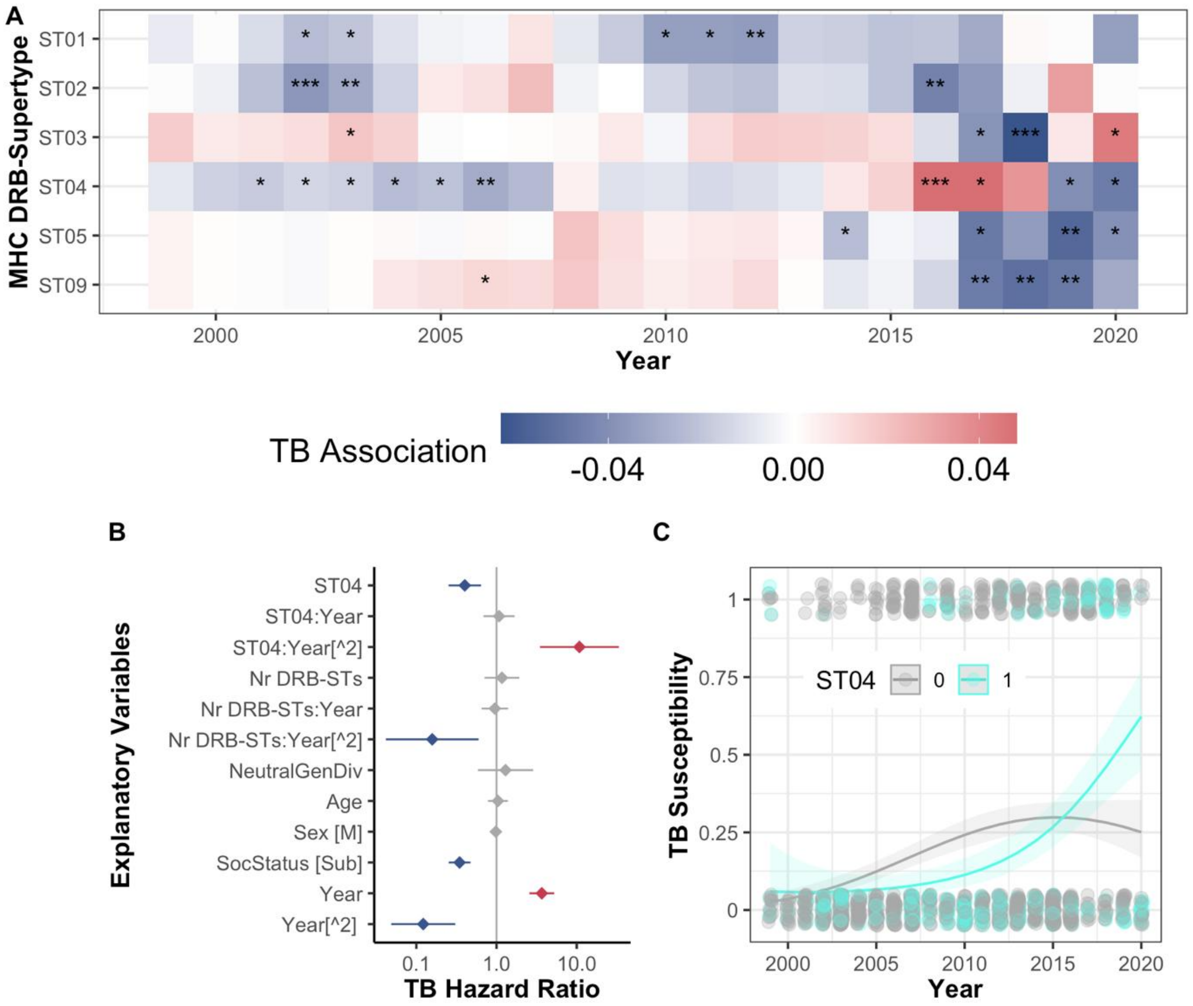

Supplementary Fig 3. MHC-DRB supertype association with TB susceptibility. (A) Heatmap displaying higher (i.e., positive association) and lower (i.e., negative association) likelihood of observing TB signs in meerkats with different MHC-DRB supertypes based on co-occurrence analysis (p-value: *<0.05, **<0.01, p*<0.001). (B) Effect sizes (± 95% CIs; n=1,497) for all explanatory variables included in generalized linear mixed effect model including the MHC ST04, its interaction with time, while controlling for biological and socio-ecological variables; Significant effects are in blue (lower likelihood of developing TB signs) and red (higher likelihood of developing TB signs). (C) Visualization of non-linear effect of ST04 (cyan) on TB over time (effect size ± 95% CIs).

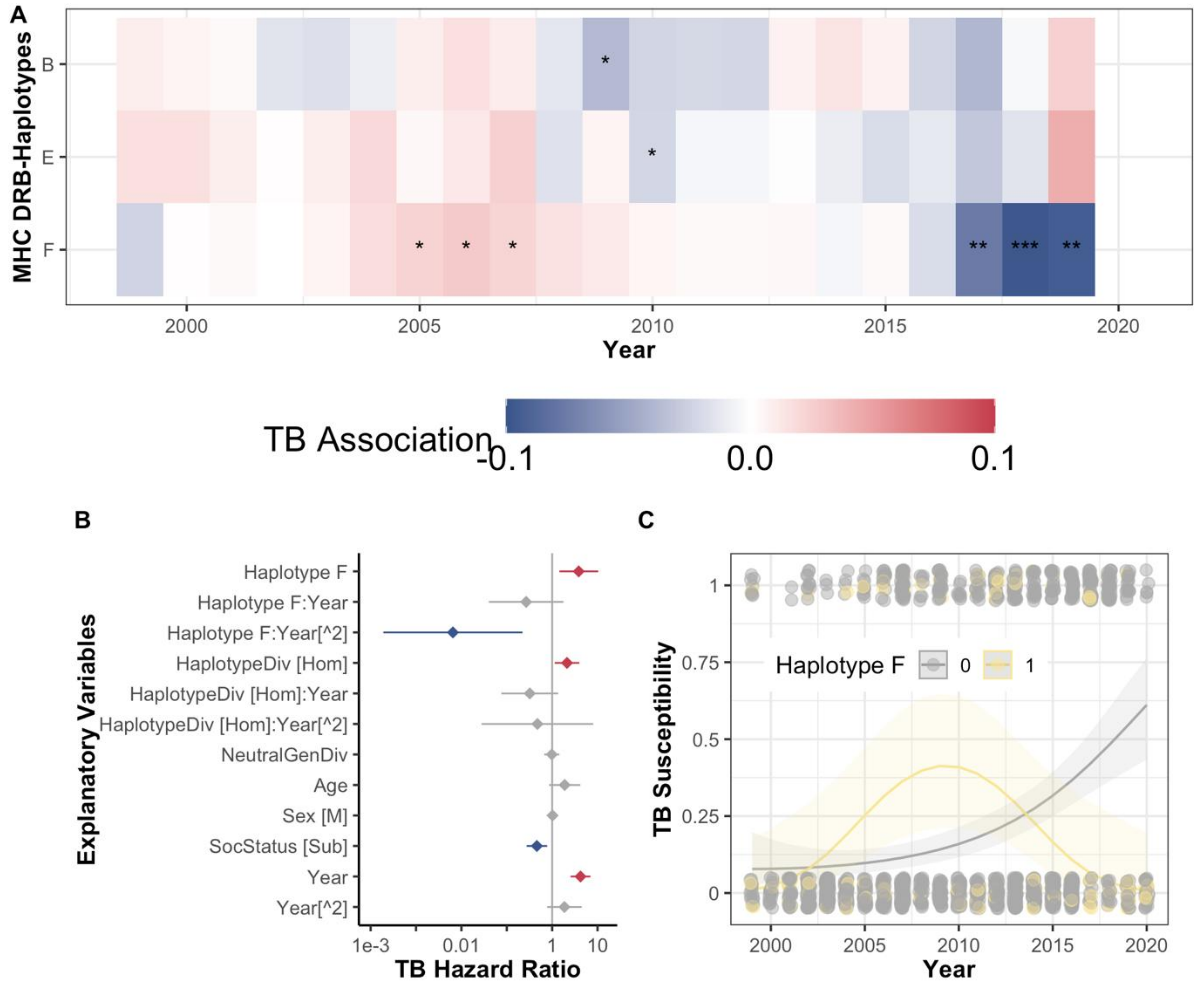

Supplementary Fig 4. MHC-DRB haplotype association with TB susceptibility. (A) Heatmap displaying higher (i.e., positive association) and lower (i.e., negative association) likelihood of observing TB signs in meerkats with different MHC-DRB haplotypes (p-value: *<0.05, **<0.01, p*<0.001). (B) Effect sizes (± 95% CIs n=714) for all explanatory variables included in generalized linear mixed effect model including the MHC haplotype F, its interaction with time, while controlling for biological and socio-ecological variables; Significant effects are in blue (lower likelihood of developing TB signs) and red (higher likelihood of developing TB signs). (C) Visualization of non-linear effect of haplotype F (yellow) on TB over time (effect size ± 95% CIs; n=714).

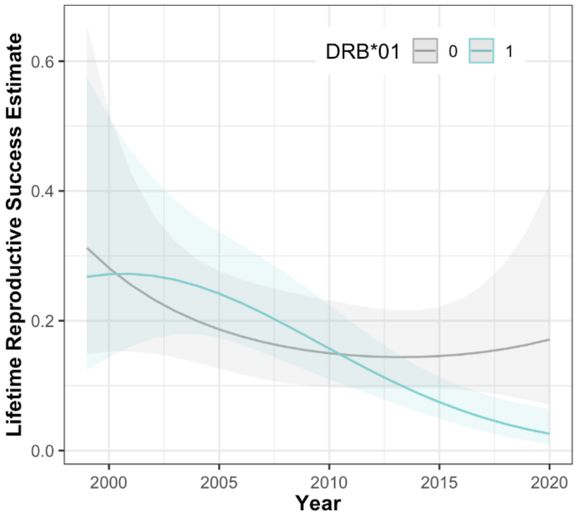

Supplementary Fig 5. Carrying MHC allele Susu-DRB*01 (turquoise) affected lifetime reproductive success (effect size ±.95% CIs; n=1,497).

Supplementary Tables

Supplementary Table 1. Mean and standard deviation of lifetime reproductive success and longevity in the complete (n=3420 individuals) and MHC-typed dataset (n=1567 individuals).

**Group**	**Dominant**	**mean LRS (+/- SD)**	**mean longevity (years) (+/- SD)**
**Females in complete dataset**	Yes	7.78 ± 12.58	4.16 ± 2.21
**Females in complete dataset**	No	0.54 ± 3.38	1.63 ± 1.30
**Females in MHC-typed dataset**	Yes	7.95 ± 12.87	4.17 ± 2.20
**Females in MHC-typed dataset**	No	0.33 ± 1.03	1.71 ± 1.11
**Males in complete dataset**	Yes	4.43 ± 8.71	4.17 ± 2.08
**Males in complete dataset**	No	0.35 ± 2.06	1.53 ± 1.26
**Males in MHC-typed dataset**	Yes	4.35 ± 8.64	4.19 ± 2.08
**Males in MHC-typed dataset**	No	0.24 ± 1.16	1.67 ± 1.08

Supplementary Table 2. Sample size distribution of MHC-typed meerkats across years. Listed are also the number of MHC-typed meerkats with clinical signs of TB in each year and the number of social groups sampled. For a graphical display see Figure 1B.

**Year**	**Total number of meerkats**	**Number of MHC-typed meerkats**	**Meerkats with TB***	**Number of social groups***
**1999**	296	139	24	11
**2000**	283	154	0	9
**2001**	364	198	4	11
**2002**	403	260	13	15
**2003**	372	255	7	13
**2004**	319	198	13	12
**2005**	388	253	25	15
**2006**	456	320	42	14
**2007**	388	286	93	16
**2008**	350	175	23	15
**2009**	433	236	61	16
**2010**	407	268	26	15
**2011**	518	342	61	14
**2012**	490	332	94	21
**2013**	378	262	68	19

**2014**	465	289	59	21
**2015**	461	266	53	24
**2016**	349	232	90	21
**2017**	283	170	90	18
**2018**	302	174	92	11
**2019**	275	135	38	11
**2020**	298	76	16	9

*of the subset of MHC-typed meerkats relevant for this study

Supplementary Table 3. Raw RNA sequencing data details

SRA ID	Individual	Tissue
SRR9024738	meerkat8	Testes
SRR9024739	meerkat8	Adrenal Gland
SRR9024740	meerkat11	Cerebellum
SRR9024741	meerkat11	Cerebrum Temporal Lobe
SRR9024744	meerkat8	Cerebrum Frontal Lobe
SRR9024745	meerkat8	Cerebellum
SRR9024746	meerkat11	Heart
SRR9024747	meerkat11	Liver
SRR9024748	meerkat11	Intestine
SRR9024749	meerkat11	Testes
SRR9024752	meerkat11	Adrenal Gland
SRR9024753	meerkat11	Kidney
SRR9024754	meerkat11	Spleen
SRR9024755	meerkat11	Lung

Supplementary Table 4. Genomic coordinates of MHC class II genes and exons in the meerkat reference assembly (GCF_006229205)

Scaffold	Start	End	Exon Name	Strand
NC_043706.1	30978973	30979061	mSurSur_DMA1_exon1	-
NC_043706.1	30977175	30977460	mSurSur_DMA1_exon2	-
NC_043706.1	30976248	30976527	mSurSur_DMA1_exon3	-
NC_043706.1	30975884	30976013	mSurSur_DMA1_exon4	-
NC_043706.1	30975536	30975541	mSurSur_DMA1_exon5	-
NC_043706.1	30967254	30967309	mSurSur_DMB1_exon1	-
NC_043706.1	30966302	30966587	mSurSur_DMB1_exon2	-
NC_043706.1	30964819	30965104	mSurSur_DMB1_exon3	-
NC_043706.1	30963283	30963400	mSurSur_DMB1_exon4	-
NC_043706.1	30963093	30963129	mSurSur_DMB1_exon5	-
NC_043706.1	30962724	30962741	mSurSur_DMB1_exon6	-
NC_043706.1	31024871	31024953	mSurSur_DOA1_exon1	-
NC_043706.1	31023450	31023699	mSurSur_DOA1_exon2	-
NC_043706.1	31022738	31023020	mSurSur_DOA1_exon3	-

NC_043706.1	31022517	31022653	mSurSur_DOA1_exon4	-
NC_043706.1	31022272	31022276	mSurSur_DOA1_exon5	-
NC_043706.1	30846113	30846204	mSurSur_DOB1_exon1	-
NC_043706.1	30842654	30842924	mSurSur_DOB1_exon2	-
NC_043706.1	30841907	30842189	mSurSur_DOB1_exon3	-
NC_043706.1	30841319	30841430	mSurSur_DOB1_exon4	-
NC_043706.1	30841033	30841065	mSurSur_DOB1_exon5	-
NC_043706.1	30840833	30840860	mSurSur_DOB1_exon6	-
NC_043706.1	31059634	31059734	mSurSur_DPA1_exon1	-
NC_043706.1	31056778	31057024	mSurSur_DPA1_exon2	-
NC_043706.1	31056118	31056400	mSurSur_DPA1_exon3	-
NC_043706.1	31055752	31055899	mSurSur_DPA1_exon4	-
NC_043706.1	31051753	31051812	mSurSur_DPA1_exon5	-
NC_043706.1	31062145	31062245	mSurSur_DPB1_exon1	+
NC_043706.1	31069139	31069409	mSurSur_DPB1_exon2	+
NC_043706.1	31070342	31070624	mSurSur_DPB1_exon3	+
NC_043706.1	31071153	31071264	mSurSur_DPB1_exon4	+
NC_043706.1	31072261	31072275	mSurSur_DPB1_exon5	+
NC_043706.1	30748806	30748888	mSurSur_DQA_exon1	+
NC_043706.1	30771959	30772068	mSurSur_DQB_exon1	-
NC_043706.1	30770299	30770569	mSurSur_DQB_exon2	-
NC_043706.1	30768372	30768654	mSurSur_DQB_exon3	-
NC_043706.1	30767747	30767858	mSurSur_DQB_exon4	-
NC_043706.1	30767239	30767259	mSurSur_DQB_exon5	-
NC_043706.1	30766625	30766639	mSurSur_DQB_exon6	-
NC_043706.1	30491349	30491431	mSurSur_DRA1_exon1	+
NC_043706.1	30493040	30493286	mSurSur_DRA1_exon2	+
NC_043706.1	30493744	30494026	mSurSur_DRA1_exon3	+
NC_043706.1	30494332	30494487	mSurSur_DRA1_exon4	+
NC_043706.1	30689508	30689608	mSurSur_DRB1_exon1	-
NC_043706.1	30684360	30684630	mSurSur_DRB1_exon2	-
NC_043706.1	30659908	30660190	mSurSur_DRB1_exon3_flipped	+
NC_043706.1	30657346	30657628	mSurSur_DRB1_exon3_Ngap	-
NC_043706.1	30656429	30656540	mSurSur_DRB1_exon4	-
NC_043706.1	30655937	30655961	mSurSur_DRB1_exon5	-
NC_043706.1	30655658	30655672	mSurSur_DRB1_exon6	-
NW_021859663.1	65330	65430	mSurSur_DRB2_exon1	-
NW_021859663.1	59655	59925	mSurSur_DRB2_exon2	-
NW_021859663.1	57629	57911	mSurSur_DRB2_exon3	-
NW_021859663.1	56484	56595	mSurSur_DRB2_exon4	-
NW_021859663.1	55575	55599	mSurSur_DRB2_exon5	-
NW_021859663.1	55269	55283	mSurSur_DRB2_exon6	-
NC_043706.1	30529684	30529781	mSurSur_DRBp1_exon1	-
NC_043706.1	30521808	30522090	mSurSur_DRBp1_exon3	-
NC_043706.1	30520951	30521062	mSurSur_DRBp1_exon4	-
NC_043706.1	30520459	30520483	mSurSur_DRBp1_exon5	-

NC_043706.1	30520150	30520164	mSurSur_DRBp1_exon6	-
NW_021859663.1	31925	32025	mSurSur_DRBp2_exon1	-
NW_021859663.1	25830	26112	mSurSur_DRBp2_exon2	-
NW_021859663.1	23986	24097	mSurSur_DRBp2_exon4	-
NW_021859663.1	23501	23525	mSurSur_DRBp2_exon5	-
NW_021859663.1	23194	23208	mSurSur_DRBp2_exon6	-
NW_021870370.1	373	655	mSurSur_DQA_exon3	+
NW_021870370.1	892	1047	mSurSur_DQA_exon4	+
NW_021877437.1	997	1279	mSurSur_DQA_exon3	-
NW_021877437.1	605	760	mSurSur_DQA_exon4	-
NW_021891667.1	162	411	mSurSur_DQA_exon2	-
NW_021891698.1	230	479	mSurSur_DQA_exon2	+
NW_021880693.1	332	599	mSurSur_DRB_exon2*	-
NW_021885817.1	114	381	mSurSur_DRB_exon2*	+

*Indicates stop codon found

Supplementary Table 5. Genes with supporting transcriptome evidence, indicating functional coding genes; Suffix ‘P’ denotes presumed pseudogenes.

	DMA1	DMB1	DOA1	DOB1	DPA1	DPB1	DQB1	DRA1	DRB1	DRB1P	DRB2	DRB2P
meerkat8_Testes	Y	Y	N	Y	N	Y	Y	Y	Y	N	N	N
meerkat8_Adrenal Gland	Y	N	N	N	N	N	N	N	Y	N	N	N
meerkat11_Cerebellum	Y	Y	N	N	N	Y	N	Y	Y	N	N	N
meerkat11_Cerebrum Temporal Lobe	Y	Y	N	N	N	N	N	Y	Y	N	Y	N
meerkat8_Cerebrum Frontal Lobe	Y	Y	N	N	N	N	N	Y	Y	N	N	N
meerkat8_Cerebellum	Y	Y	N	N	N	N	N	Y	Y	N	N	N
meerkat11_Heart	Y	Y	N	N	Y	N	N	Y	Y	N	Y	N
meerkat11_Liver	Y	Y	N	N	Y	N	N	Y	Y	N	Y	N
meerkat11_Intestine	Y	Y	Y	Y	N	Y	Y	Y	Y	N	Y	N
meerkat11_Testes	Y	Y	N	Y	N	N	Y	Y	Y	N	Y	N
meerkat11_Adrenal Gland	Y	Y	N	N	N	N	N	Y	Y	N	Y	N
meerkat11_Kidney	Y	Y	N	N	N	N	N	Y	Y	N	Y	N
meerkat11_Spleen	N	N	N	N	N	N	N	Y	Y	N	Y	N

meerkat11_Lung	Y	Y	Y	Y	Y	Y	Y	Y	Y	N	Y	N
No. Tissues Expressed	13	12	2	4	3	4	4	13	14	0	9	0

Supplementary Table 6. Results from a linear regression on pairwise estimated Fst with temporal distance (i.e., years between Fst measure) and whether the Fst was calculated from MHC or microsatellite data as explanatory variable. Significant effects are given in bold.

**Coefficients**	**Estimate**	**Std. Error**		**p-value**
Intercept	-1.2×10^{-6}	3.8×10^{-4}		0.998
Year distant	1.9×10^{-3}	4.1×10^{-5}		**<0.001**
MHC vs. Microsat	1.0×10^{-3}	5.4×10^{-4}		0.059
Interaction	-7.8×10^{-4}	5.9×10^{-5}		**<0.001**

Supplementary Table 7. Complete list of MHC-Supertypes with corresponding MHC-DRB-alleles

**Supertype**	**Assigned alleles**
**ST01**	DRB*01, DRB*41, DRB*56
**ST02**	DRB*03, DRB*06, DRB*31, DRB*38, DRB*39, DRB*43, DRB*47, DRB*50, DRB*59
**ST03**	DRB*07, DRB*20
**ST04**	DRB*09, DRB*16, DRB*25
**ST05**	DRB*10, DRB*26, DRB*58
**ST06**	DRB*12, DRB*29, DRB*42, DRB*48, DRB*65
**ST07**	DRB*17, DRB*18, DRB*37
**ST08**	DRB*11, DRB*32, DRB*55, DRB*62
**ST09**	DRB*13
**ST10**	DRB*14
**ST11**	DRB*36, DRB*68
**ST12**	DRB*35

Supplementary Table 8. Complete list of MHC-haplotypes with corresponding MHC-DRB alleles, and whether they contained non-functional alleles with premature stop codons (i.e., pseudogenes).

Haplotype	Assigned alleles	Functional?
**A**	DRB*02	NO
**B**	DRB*01, DRB*03	**YES**
**C**	DRB*04	NO
**D**	DRB*05, DRB*09	NO
**E**	DRB*01, DRB*06	**YES**
**F**	DRB*01, DRB*10, DRB*13	**YES**
**G**	DRB*02, DRB*11, DRB*12	**YES**
**H**	DRB*05, DRB*17	NO
**I**	DRB*01	**YES**
**J**	DRB*03	**YES**
**K**	DRB*04, DRB*16	NO
**L**	DRB*05, DRB*18, DRB*20	NO
**M**	DRB*04, DRB*20	NO
**N**	DRB*01, DRB*10	**YES**
**O**	DRB*05, DRB*16	NO
**P**	DRB*05, DRB*18	NO
**Q**	DRB*06	**YES**
**R**	DRB*05	NO
**S**	DRB*01, DRB*03, DRB*06	**YES**
**T**	DRB*09	**YES**
**U**	DRB*16	**YES**
**V**	DRB*10, DRB*13	**YES**
**W**	DRB*01, DRB*02	NO
**X**	DRB*02, DRB*09	NO
**Y**	DRB*01, DRB*04	NO
**Z**	DRB*05, DRB*13, DRB*18	NO
**AA**	DRB*11, DRB*12	**YES**
**AB**	DRB*01, DRB*11	**YES**
**AC**	DRB*01, DRB*04	NO
**AD**	DRB*17	**YES**
**AE**	DRB*02, DRB*11	NO
**AF**	DRB*11	**YES**
**AG**	DRB*02, DRB*12	NO

Supplementary Table 9 - 14: Model summary tables for all susceptibility, progression, resilience, mortality and lifetime reproductive success models.

See attached excel sheet

Supplementary Data files

Supplementary Data 1. Amino-acid sequence alignment of all reliably identified alleles for *Suricata suricatta*. *See attached excel sheet*
