# Supplementary material for: Twenty years of tuberculosis-driven selection shaped the evolution of the meerkat major histocompatibility complex
Source: Nat Ecol Evol. 2025 Aug 25;9(11):2161–72. doi: 10.1038/s41559-025-02837-x (PMC12592209; doi:10.1038/s41559-025-02837-x)
Supplement: Supplementary file 2 — Reporting Summary [file 41559_2025_2837_MOESM2_ESM.pdf]

nature portfolio

Corresponding author(s): Nadine Müller-Klein

Last updated by author(s): Jul 9, 2025

Reporting Summary

Nature Portfolio wishes to improve the reproducibility of the work that we publish. This form provides structure for consistency and transparency in reporting. For further information on Nature Portfolio policies, see our Editorial Policies and the Editorial Policy Checklist.

Statistics

For all statistical analyses, confirm that the following items are present in the figure legend, table legend, main text, or Methods section.

n/a	Confirmed	
☐	☒	The exact sample size (n) for each experimental group/condition, given as a discrete number and unit of measurement
☐	☒	A statement on whether measurements were taken from distinct samples or whether the same sample was measured repeatedly
☐	☒	The statistical test(s) used AND whether they are one- or two-sided<br>*Only common tests should be described solely by name; describe more complex techniques in the Methods section.*
☐	☒	A description of all covariates tested
☐	☒	A description of any assumptions or corrections, such as tests of normality and adjustment for multiple comparisons
☐	☒	A full description of the statistical parameters including central tendency (e.g. means) or other basic estimates (e.g. regression coefficient) AND variation (e.g. standard deviation) or associated estimates of uncertainty (e.g. confidence intervals)
☐	☒	For null hypothesis testing, the test statistic (e.g. F, t, r) with confidence intervals, effect sizes, degrees of freedom and P value noted<br>*Give P values as exact values whenever suitable.*
☒	☐	For Bayesian analysis, information on the choice of priors and Markov chain Monte Carlo settings
☒	☐	For hierarchical and complex designs, identification of the appropriate level for tests and full reporting of outcomes
☐	☒	Estimates of effect sizes (e.g. Cohen's d, Pearson's r), indicating how they were calculated

Our web collection on statistics for biologists contains articles on many of the points above.

Software and code

Policy information about availability of computer code

Data collection	Behavioural data on meerkats was collected using pendragon; Data was extracted from the Meerkat Database in June 2023; Database: MySQL
Data analysis	Bioinformatic and statistical analyses were performed in the software R (v4.2.3/ 4.3.2; R Core Team 2022). The R code including all packages used for the analyses of the current study will be made available on FigShare prior to publication, links to FigShare are provided with the submission.

For manuscripts utilizing custom algorithms or software that are central to the research but not yet described in published literature, software must be made available to editors and reviewers. We strongly encourage code deposition in a community repository (e.g. GitHub). See the Nature Portfolio guidelines for submitting code & software for further information.

Data

Policy information about availability of data

All manuscripts must include a data availability statement. This statement should provide the following information, where applicable:

- Accession codes, unique identifiers, or web links for publicly available datasets
- A description of any restrictions on data availability
- For clinical datasets or third party data, please ensure that the statement adheres to our policy

The dataset generated and/or analyzed during the current study will be made available on Figshare permanently pending acceptance. https://doi.org/10.6084/m9.figshare.26172985

Research involving human participants, their data, or biological material

Policy information about studies with human participants or human data. See also policy information about sex, gender (identity/presentation), and sexual orientation and race, ethnicity and racism.

Reporting on sex and gender	N/A
Reporting on race, ethnicity, or other socially relevant groupings	N/A
Population characteristics	N/A
Recruitment	N/A
Ethics oversight	N/A

Note that full information on the approval of the study protocol must also be provided in the manuscript.

Field-specific reporting

Please select the one below that is the best fit for your research. If you are not sure, read the appropriate sections before making your selection.

☐ Life sciences ☐ Behavioural & social sciences ☒ Ecological, evolutionary & environmental sciences

For a reference copy of the document with all sections, see nature.com/documents/nr-reporting-summary-flat.pdf

Ecological, evolutionary & environmental sciences study design

All studies must disclose on these points even when the disclosure is negative.

Study description	Data was collected within the Kalahari Meerkat Project, with data on known individuals collected since 1993. Life history data (births, deaths/ dissappearances, reproductive events), social group, group change events and clinical signs of Mycobacterium suricattae infections (TB) were recorded longitudinally on an individual level on an almost daily basis. Tissue samples for genetics were collected early in life from each individual born into the study group. Tissue samples were taken in from of a tail clip when meerkats were marked early in life. All other data used for this study was collected non-invasively.<br>DNA had been extracted from tissue samples at the Universities of Edinburgh and Zurich. Microsatellite sequencing was completed at the University of Zürich. Aliquots were taken to Ulm University for MHC genotyping of the MHC-DRB locus via high throuput sequencing. Due to the longitudinal nature of the study, we could assess the effect of individual MHC-genotype on TB susceptibility (lifetime likelihood to develop signs of TB), TB resilience (time surviving after first signs of TB), progression (likelihood to progress to TB within 3 months) and overall mortality (likelihood to die within 3 months). Simultaneously, we could assess the link between frequency of specific MHC alleles and TB prevalence, testing for evidence for negative frequency-dependent selection as the mechanisms of pathogen mediated selection on wild meerkat MHC.
Research sample	Within the study population of wild, habituated meerkats, individuals from groups persisting for longer than 2 years with on average more than 6 resident individuals and a history of clinical infections with Mycobacterium suricattae were targeted for MHC-genotyping. Additionally, groups with no record of M. suricattae infections were targeted as controls. Our sampling regime resulted in 1567 successfully genotpyed individuals.<br>Observational data and metadata for the study animals were extracted fom the Kalahari Meerkat Project Database. Tissue samples (tail tips) had been collected from individuals shortly after first emergence from the burrow and extracted DNA from the samples was utilized for the study.
Sampling strategy	Tissue samples were systematically collected, aiming at samples from all individuals within the study population. Within the Kalahari Meerkat Project, collection of observational data was distributed as evenly as possible across the study groups within the Project, with almost daily visits to each study group.
Data collection	TCB and MM organized the fieldwork, data management and sample collection, beginning in 1993. Data and samples were collected by a number of researchers and field assistants over the course of the project. KW, NMK and VR conducted lab work for MHC-genotpying, NMK, DWM and VR analysed the data.
Timing and spatial scale	Data collection within the Kalahari Meerkat Project has been ongoing from 1993 to date. For our study, we focussed on individuals born by 2020 and analysed data collected between 1999 and 2020. Data was collected from all habituated groups within the Kuruman Research Centre in Northern South Africa.
Data exclusions	Samples with less than 10.000 blasted reads were excluded from further analyses.<br>Animals were excluded from analyses if metadata was incomplete (no information on sex, microsatellite heterozygosity). One individual carrying only one MHC-DRB allele with a premature stop Codon was excluded from further analyses. For linear modelling analyses, individuals that died before 1999 or after 2020 were excluded, reducing the dataset from 1566 individuals to 1497 for MHC-dataset and 714 individuals for the haplotyped dataset.

	For survial analyses, individuals with missing information and individual periods before 1999 and after 2020 were excluded, resulting in a final sample size of 1535 individuals.
Reproducibility	The extensive fieldwork that resulted in an enormous data set during the course of this study, allowing us to analyse almost half of the studied population of over 3400 individuals, spanning multiple social groups within an area of ~ 50 km^2
Randomization	Due to longitudinal observational data collection, randomization is not applicable to the studied indiviuals.<br>Individuals were assigned to social groups based on observed presence in the respective social groups. Infection status was determined based on clinical signs of infection for every individual on an almost daily basis.
Blinding	All laboratory work was performed blind to the identity of the sample.

Did the study involve field work? ☒ Yes ☐ No

Field work, collection and transport

Field conditions	The Kuruman River Reserve covers around 50km2 of semi-arid dune country on either side of the dry bed of the Kuruman River in the South African Kalahari. The topography of the reserve consists of vegetated ("fossil") dunes covered with grasses and separated by valleys up to 300 m across. The dunes flatten as they approach the bed of the Kuruman River, which is usually dry. Vegetation consists of scattered camel thorn trees (Acacia erioloba) along the river bed, grading out into dry scrub dominated by annual and perennial grasses and Rhigozum scrub. There is around 250 mm/10 in of rain a year, mostly falling between October and March. In the summer (October-March), temperatures range from midday highs in the low 40s°C (November-December) to night-time lows of around 20°C. Temperatures are lower in winter (April-September) and often fall below 0°C at night in the coldest month (July). Humidity is typically very low.
Location	The Kalahari Research Centre is situated in the Kuruman River Reserve (KRR), in the Northern Cape, South Africa approximately 17 km south of the Botswana border and 30km west of the small town of Vanzylsrus. GPS coordinates: 26°58'S, 21° 49'E
Access & import/export	Samples utilized for the current study were already available from University of Zürich, where import/ export permits had previously been obtained.
Disturbance	Wild meerkats were carefully habituated and are familiar with the researchers, causing minimal disturbance to the population.

Reporting for specific materials, systems and methods

We require information from authors about some types of materials, experimental systems and methods used in many studies. Here, indicate whether each material, system or method listed is relevant to your study. If you are not sure if a list item applies to your research, read the appropriate section before selecting a response.

Materials & experimental systems

n/a	Involved in the study	
☒	☐	Antibodies
☒	☐	Eukaryotic cell lines
☒	☐	Palaeontology and archaeology
☐	☒	Animals and other organisms
☒	☐	Clinical data
☒	☐	Dual use research of concern
☒	☐	Plants

Methods

n/a	Involved in the study	
☒	☐	ChIP-seq
☒	☐	Flow cytometry
☒	☐	MRI-based neuroimaging

Animals and other research organisms

Policy information about studies involving animals; ARRIVE guidelines recommended for reporting animal research, and Sex and Gender in Research

Laboratory animals	The study did not involve laboratory animals.
Wild animals	Wild meerkats were observed on an almost daily basis, allowing for collection of detailed life-history data, including date of birth and death/ dissappearance. Individuals of all ages were included and data on their age, TB status and resident group were available.
Reporting on sex	Sex was included in all analyses as a control variable. Individuals were sexed based on external sexual characteristics (presence of testes). Of 1566 genotyped individuals, 701 were identified as female, 837 as male, and 28 did not have sex assignement (died as pups before sex determination) and were excluded from further analyses.
Field-collected samples	Tissue samples for genetic analyses were collected by cutting a small piece of the tail (~2 mm) from<br>pups at first emergence from the burrow and from adult individuals when they immigrated into the study population.

	Sampled individuals returned to normal behaviour shortly after sampling and no infections of cuts were reported. There is no evidence for any short or long-term negative consequences of sampling the meerkats. Samples were stored in DMSO or 100 mm EDTA 95% ethanol at –20 °C. Extraction prior to 2002 was performed using standard chelex or phenol/chloroform methods. From 2002 on DNeasy Blood and Tissue kits by Quiagen were used for DNA extraction. Extracted samples were stored in double-distilled water at –20 °C, aliquots were transported to Ulm on ice.
Ethics oversight	Behavioural data and sample collection for this study was conducted with permission of the ethical committee of 383 Pretoria University and the Northern Cape Conservation Service, South Africa (Permit 384 number: EC031-13, FAUNA 1020-2016)

Note that full information on the approval of the study protocol must also be provided in the manuscript.

Plants

Seed stocks	N/A
Novel plant genotypes	N/A
Authentication	N/A
